# Intracoronary Imaging of Vulnerable Plaque—From Clinical Research to Everyday Practice

**DOI:** 10.3390/jcm11226639

**Published:** 2022-11-09

**Authors:** Jacek Legutko, Krzysztof L. Bryniarski, Grzegorz L. Kaluza, Tomasz Roleder, Elzbieta Pociask, Elvin Kedhi, Wojciech Wojakowski, Ik-Kyung Jang, Pawel Kleczynski

**Affiliations:** 1 Department of Interventional Cardiology, Faculty of Medicine, Institute of Cardiology, Jagiellonian University Medical College, 31-202 Kraków, Poland; 2 Clinical Department of Interventional Cardiology, John Paul II Hospital, 31-202 Kraków, Poland; 3 Skirball Center for Innovation, Cardiovascular Research Foundation, Orangeburg, NY 10019, USA; 4 Department of Cardiology, Wroclaw Medical University, 50-556 Wroclaw, Poland; 5 Department of Biocybernetics and Biomedical Engineering, AGH University of Science and Technology, 30-059 Kraków, Poland; 6 Clinique Hopitaliere Erasme, Université Libre de Bruxelles, 1070 Brussels, Belgium; 7 Division of Cardiology and Structural Heart Diseases, Medical University of Silesia, 40-635 Katowice, Poland; 8 Cardiology Division, Massachusetts General Hospital, Harvard Medical School, GRB 800, Boston, MA 02115, USA; 9 Division of Cardiology, Kyung Hee University Hospital, Seoul 02447, Korea

**Keywords:** cardiovascular disease, acute myocardial infarction, intravascular imaging, vulnerable coronary plaque

## Abstract

The introduction into clinical practice of intravascular imaging, including intravascular ultrasound (IVUS), optical coherence tomography (OCT) and their derivatives, allowed for the in vivo assessment of coronary atherosclerosis in humans, including insights into plaque evolution and progression process. Intravascular ultrasound, the most commonly used intravascular modality in many countries, due to its low resolution cannot assess many features of vulnerable plaque such as lipid plaque or thin-cap fibroatheroma. Thus, novel methods were introduced to facilitate this problem including virtual histology intravascular ultrasound and later on near-infrared spectroscopy and OCT. Howbeit, none of the currently used modalities can assess all known characteristics of plaque vulnerability; hence, the idea of combining different intravascular imaging methods has emerged including NIRS-IVUS or OCT-IVUS imaging. All of those described methods may allow us to identify the most vulnerable plaques, which are prone to cause acute coronary syndrome, and thus they may allow us to introduce proper treatment before plaque destabilization.

## 1. Introduction

Ischemic heart disease (IHD) remains one of the most common causes of death in developed countries. The most frequent pathology behind IHD is coronary artery disease (CAD) caused by atherosclerosis. Clinical manifestations of CAD include sudden cardiac death (SCD), acute coronary syndromes (ACS: ST-segment elevation myocardial infarction (STEMI), non-ST-segment elevation myocardial infarction (NSTEMI), unstable angina) and chronic coronary syndromes. Acute myocardial ischemia responsible for SCD and ACS is usually associated with a rapid progression of high-risk coronary atherosclerotic plaque (vulnerable plaque) caused by rupture, erosion or ulceration followed by intracoronary thrombus formation. Acute obstruction of coronary artery as a cause of SCD was first described in the year 1912 by James B. Herrick [1]. The introduction into clinical practice of intravascular imaging, including intravascular ultrasound (IVUS), optical coherence tomography (OCT) and their derivatives, allowed for the in vivo assessment of coronary atherosclerosis in humans, including the mechanisms responsible for SCD and ACS, as well as a serial assessment of the mechanisms and risk factors for coronary atherosclerotic plaque progression and destabilization. Using those methods in everyday clinical practice, we are now able to detect atherosclerotic plaques, which are at high risk of rapid progression leading to subsequent coronary events. Next, clinical studies have to investigate if the mechanical stabilization of high-risk intravascular-imaging-derived vulnerable plaque that do not cause myocardial ischemia will decrease the risk of major adverse cardiac events at follow-up in comparison to optimal medical therapy. The aim of this article is to summarize the current knowledge on different intravascular imaging methods of coronary vulnerable plaque assessment and their application in ongoing research studies and in everyday clinical practice.

## 2. Vulnerable Plaque and Its Visualization

### 2.1. Pathology of Vulnerable Plaque

The inability to predict which atherosclerotic lesions will become the source of clinical instability and a culprit lesion in ACS has long remained the Achilles’ heel of cardiovascular medicine. Despite groundbreaking advances in preventive cardiology and the interventional treatment of atherosclerosis leading to a decreased burden of cardiovascular disease worldwide, millions of patients experience life-altering fatal coronary events without any prior warning, even despite what is believed to be optimal medical therapy. Insights gained from the pathological examination of coronary arteries from victims of fatal coronary events reveal that many atherosclerotic plaques appear to progress through multiple cycles of necrotic core expansion, intraplaque hemorrhage and ruptures, and healing [2,3]. Although most of these transformations involve some form of intracoronary (intraplaque or intraluminal) thrombus development, they do not necessarily lead to anginal symptoms and/or coronary occlusion [4]. Lesions may remain clinically silent for years until one of these transformation cycles finally produces symptoms, often in the most dramatic form of myocardial infarction and/or death.

**Figure 1:**
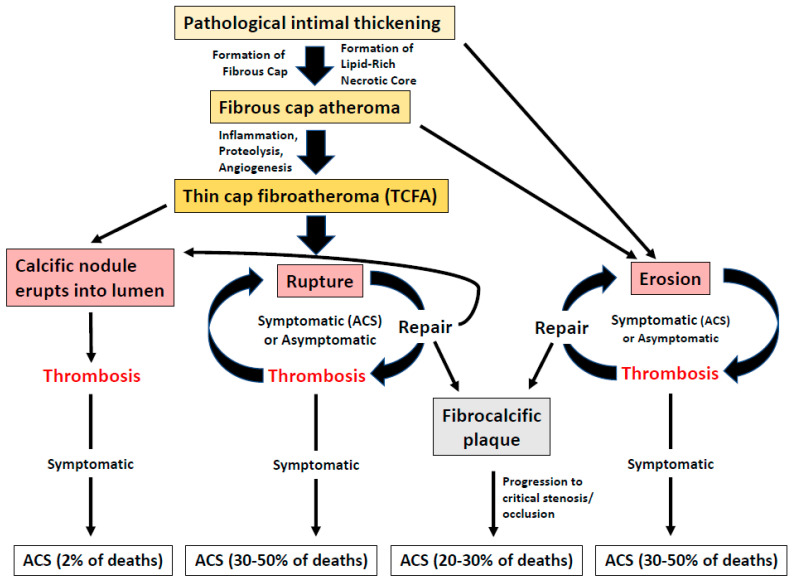
Key insights into plaque evolution and progression processes, and their clinical correlates.

Figure 1 summarizes the key insights into plaque evolution and progression processes, and their clinical correlates. Pathological intimal thickening (PIT) is the most accepted lesion precursor of complex fibro-atheroma formation. The transition from PIT to atherosclerotic plaque is most commonly defined by the appearance of lipid pool [5,6]. Further plaque evolution is marked by an aggressive inflammatory process often leading to core necrosis and thinning of the fibrous cap separating the increasingly morphologically unstable core from the lumen. This form of atherosclerotic plaque, termed thin-cap fibroatheroma (TCFA), was initially found most frequently in pathology specimens obtained from victims of fatal ACS [6,7]. Typical morphology would consist of large necrotic core and a cap of 65 microns or thinner infiltrated by macrophages and lymphocytes and featuring type I collagen with few or absent smooth muscle cells. Such morphology is believed to predispose to cap rupture under stress, leading to expulsion of the necrotic and lipid content of the core into the lumen and causing instant intracoronary thrombosis, often totally occlusive. Therefore, TCFA pathomorphology was initially synonymous with the clinical entity of vulnerable plaque. However, further research revealed that there are other plaque morphologies found consistently in ACS patients [8]. In addition to TCFA, there is increasing evidence, aided by growing utilization of in vivo endovascular imaging, that many ACS are triggered by surface plaque erosion [9]. Other less common forms of plaque morphologies found in ACS patients are fully or near-fully occlusive fibrocalcific plaque and erupted calcific nodule [10].

These advances led to a reformulation of the vulnerable plaque definition to a broader clinico-pathologic definition derived from currently available knowledge and recognizing retrospective and prospective aspects. As such, vulnerable plaque is now defined as any thrombosis-prone plaque or plaque at a risk of rapid progression, with potential for becoming a culprit lesion and triggering an ACS independent of its specific morphology. Furthermore, pathology research also suggests that different lesions progress differently in the same patient and can transition from the phenotype associated with clinical instability (e.g., TCFA) to the one associated with clinical stability (e.g., fibrocalcific plaque without rapid progression and/or flow-limiting stenosis), and the other way around.

### 2.2. Intravascular Ultrasound (GS-IVUS, VH-IVUS, HD-IVUS)

#### 2.2.1. Grey Scale Intravascular Ultrasound (GS-IVUS)

The introduction of GS-IVUS in the late 1990s allowed for the first time for in vivo visualization of coronary artery walls with a unique ability to assess plaque burden and distribution of the atherosclerotic plaque—otherwise not possible with the coronary angiography alone [11,12]. Pathologic studies at that time showed that the extent of the coronary atherosclerosis may be underestimated by visual analysis of angiographically normal coronary arteries. However, only after the introduction of GS-IVUS could new large scale studies in vivo verify that finding [13]. This phenomenon was explained by dilatation of the arterial wall as a response to accumulated atherosclerotic plaque—known as positive remodeling. Subsequent studies found that positive remodeling and larger plaque areas were associated with unstable coronary artery disease [14]. Moreover, gray-scale images provided by GS-IVUS allowed for the differentiation of multiple tissues: soft (with echogenicity lower than nearby adventitia), fibrous (with intermediate echogenicity), calcified (with echogenicity greater with nearby adventitia) and mixed (with several of the described acoustic signals) [11,15]. However, both its low spatial resolution and its grayscale representation did not allow GS-IVUS to be used for the identification of lipid-rich plaque and detailed analysis of plaque morphology such as visualization of TCFA—hallmarks of the vulnerable plaque [16]. One of the studies demonstrated that the positive prediction value of IVUS in detecting TCFA was 19% [17]. More importantly, IVUS ability to identify intracoronary thrombus remained limited. Hence, autoregressive spectral analysis was applied to IVUS radiofrequency backscatter data in order to facilitate the interpretation of the images of different tissue components [18,19].

#### 2.2.2. Virtual Histology Intravascular Ultrasound (VH-IVUS)

**Figure 2:**
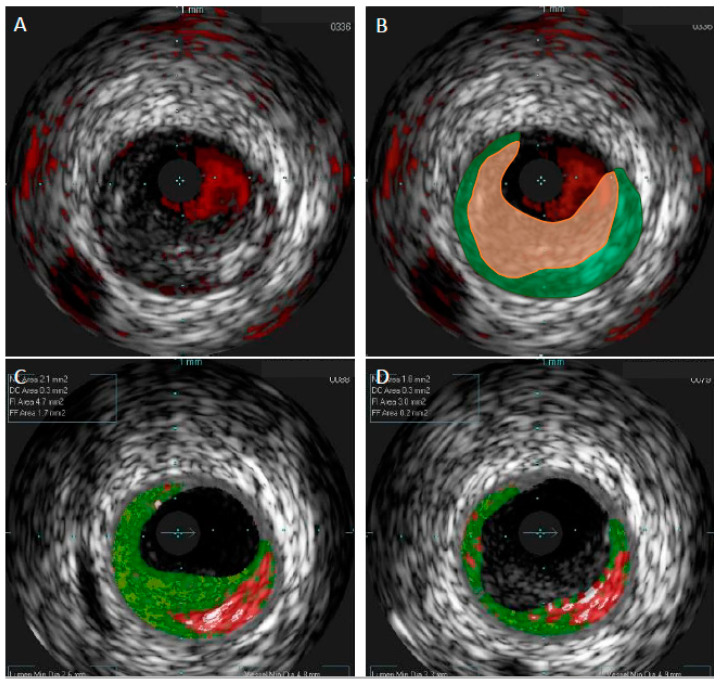
Comparison of gray-scale intravascular ultrasound and virtual histology intravascular ultrasound in patient with acute coronary syndrome. In gray-scale intravascular ultrasound (GS-IVUS), cross-section with lumen narrowing could be interpreted as soft plaque (**A**). However, on live image, motion and oscillation of the “plaque” were visible—image typical for thrombus. After postprocessing, thrombus (orange zone) was separated from true plaque (green zone) in GS-IVUS (**B**). In the same patient virtual histology intravascular ultrasound (VH-IVUS) marked thrombus as fibrotic plaque (**C**). Only after postprocessing could visualization of real borders of the plaque be presented (**D**).

Color coding of the four histological tissue subtypes in VH-IVUS allowed for a swift recognition of the plaque composition: fibrous (green); fibrofatty (yellow); necrotic core (red); and calcified (white) [20]. Histological validation demonstrated satisfactory accuracy for this technique ranging between 79.7% and 96.5% depending on the tissue type and study [19,21]. Importantly, the introduction of VH-IVUS allowed for the indirect detection of TCFA. Although VH-IVUS did not have a resolution high enough for direct imaging of TCFA, the definition of VH-TCFA was proposed in order to facilitate this problem. Presence of ≥10% of necrotic core volume without overlying fibrous tissue and with plaque burden ≥40% for three consecutive frames, which was defined as VH-TCFA, has been shown to reliably identify TCFA, as assessed by the histopathology in a study by Brown et al. [22]. However, in the same study, eight TCFAs on histopathology were not identified by VH-IVUS properly—seven of them were classified as thick-cap fibroatheromas. The authors concluded that VH-IVUS can identify large areas of NC, but it has difficulties with discriminating thin fibrous caps. It should also be emphasized that, like GS-IVUS, VH-IVUS cannot detect thrombus in coronary arteries. Furthermore, thrombus may be mistaken as fibrotic or fibrofatty plaque by VH-IVUS (Figure 2).

One of the first direct links in in vivo studies between TCFA as a feature of plaque vulnerability and subsequent adverse events was presented in prospective PROSPECT and VIVA studies, which used three-vessel imaging to assess VH-IVUS efficacy in detecting non-culprit lesions that were more likely to evolve and cause cardiovascular events [23,24]. A PROSPECT study published in 2011 remains up to date the biggest prospective study using VH-IVUS. Out of 697 patients recruited in this study, a total of 313 patients had TCFA in 596 angiographically mild (non-culprit) lesions. During the follow-up (median 3.4 years), the rate of primary endpoint, defined as a composite of death from cardiac causes, cardiac arrest, myocardial infarction or rehospitalization due to unstable or progressive angina, was 20.4%. In multivariate analysis, authors found that plaque burden ≥70%, TCFA and minimal lumen area ≤4.0 mm^2^ were independent predictors of non-culprit lesion related major adverse cardiac events in lesion-level analysis. Importantly, the rate of MACE increased from HR 3.90 (95% CI, 2.25–6.76) with TCFA alone to HR 11.05 (95% CI, 4.39–27.82) when combining all of the described plaque futures. Similarly, in a VIVA study, both large plaque burden and TCFA were predictors of non-culprit lesion related adverse events during mean 1.7 years follow-up. More recently, an ATHEROREMO-IVUS study showed that the presence of TCFA in a non-culprit coronary artery is associated with a greater incidence of death and ACS at 1 year follow-up [25]. Of note, unlike the PROSPECT and VIVA studies, in the ATHEROREMO study only single non-stenotic segment was imagined. Intriguingly, the continuation of the ATHEROREMO study published 4 years later with a long term follow-up with median of 4.7 years showed that only small lumen area and large plaque burden and not compositional plaque features on their own could predict adverse cardiovascular events in patients with coronary artery disease [26]. The authors suspected that several factors might have influenced this finding. First, there may be a change in components of atherosclerotic plaque over time—some plaques may advance to more vulnerable, whereas some may downgrade to more stable. This finding was also described by Kubo et al. in a study in which 75% of TCFA identified by VH-IVUS resolved during 12 month follow-up [27]. Second, in the ATHEROREMO study only a small part of the coronary artery tree was imagined. This problem was first raised by PROSPECT study investigators when only 52% of all non-culprit lesion related MACE arose from imagined segments.

It is not without significance that, with the use of IVUS and VH-IVUS, scientists tried to understand not only the progression but also the healing of vulnerable plaque. However, due to the limitations of those modalities, the results were mixed. A SATURN study showed a decrease in fibrofatty plaque with statin therapy, whereas a meta-analysis of several other studies showed a decrease in the necrotic core [28,29]. A GLAGOV trial showed a reduction in coronary plaque volume in patients administered with evelocumab [30]. Nevertheless, in group of patients who underwent coronary imaging with VH-IVUS, no significant differences in plaque components were observed. This finding led to the conclusion that the analysis of plaque composition by VH-IVUS may not be helpful when assessing lipid-modifying therapies. However, such a conclusion stands in contrast to findings that VH-IVUS may predict future non-culprit events by analysis of TCFA and lipid-rich plaque. One of the reasons is that VH-IVUS may not be able to discriminate the components of TCFA which mostly contribute to plaque rupture. Further explanation of this important issue was raised in an editorial by Stone et al. [2].

The introduction of IVUS not only allowed us to better understand the features of vulnerable plaque but also improved our understanding of how vulnerable plaques should be treated in terms of PCI. Legutko et al. showed that, in patients presenting with STEMI and NSTEMI, in about 50% of the patients who undergo PCI of the infarct-related artery with angiography alone, the stents do not fully cover the necrotic core site related to culprit lesions [31,32,33]. Of note, not only TCFA, small lumen area and plaque burden were suspected as features of plaque vulnerability. Amano et al., in a VH-IVUS study on 140 consecutive patients, showed that plaques with spotty or intermediate calcification without angiographic calcification were more vulnerable as opposed to plaques with angiographic calcification [34]. Lastly, with the use of IVUS, it has been shown that endothelial sheer stress may predict coronary plaque progression [35].

In summary, GS-IVUS and then VH-IVUS allowed for the first time for the in vivo visualization of coronary plaques in daily clinical practice. However, GS-IVUS, due to its limited resolution, could not provide sufficient data regarding plaque vulnerability. This problem was partially overcome with GS-IVUS, which could indirectly visualize TCFA and necrotic core. Numerous studies with the use of VH-IVUS identified factors connected to plaque progression such as TCFA, plaque burden or MLA (Table 1). However, there were still many disadvantages of both GS and VH IVUS, including low resolution or difficulties with thrombus identification; hence, the need for even better intravascular modalities emerged.

### 2.3. Near-Infrared Spectroscopy (NIRS)

**Figure 3:**
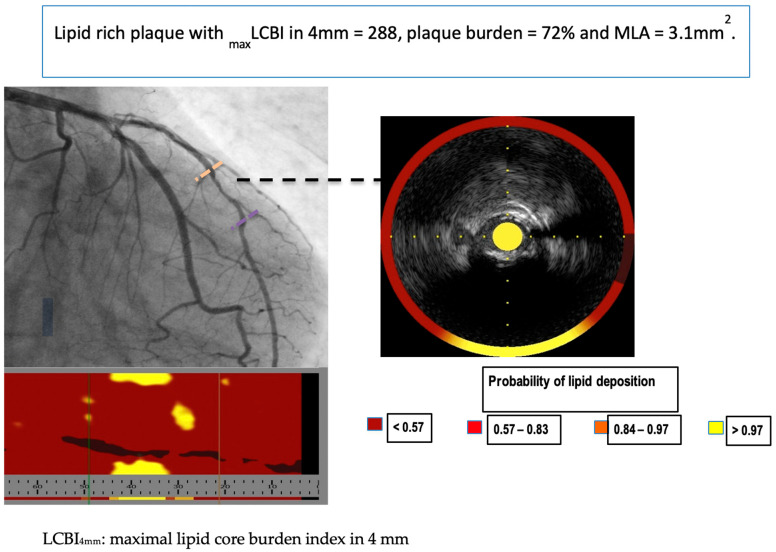
Representative image of near-infrared spectroscopy and intravascular ultrasound (NIRS-IVUS) of lipid-rich plaque.

Although VH-IVUS allowed for more detailed identification of plaque composition as compared to GS-IVUS, the need for even more detailed evaluation of coronary lesions prompted the introduction of a new modality—near-infrared spectroscopy (NIRS). Spectroscopic analysis of the backscattered light emitted by an NIR probe provides information about the cholesterol content in the arterial wall [39]. The analysis of NIR is presented as a chemogram—a color-coded map showing the probability of the presence of the lipid-rich plaque with the yellow color meaning the highest probability (≥98%) [20]. A measure of the lipid burden in the atherosclerotic plaque is provided as lipid core burden index (LCBI): number of yellow pixels on the chemogram divided by all pixels and then multiplied by 1000 (Figure 3).

Importantly, NIRS has the ability to penetrate through the calcified plaque and previously implanted stents. This new technology allowed for the overcoming of the limitations of VH-IVUS in terms of plaque lipid content evaluation [40]. One of the first studies examining vulnerable plaque with NIRS technology showed that patients with greater than median LCBI (>43.0 units) had a greater cumulative rate of all-cause mortality, stroke, non-fatal ACS and unplanned PCI during 1 year follow-up compared to patients with LCBI below median value (4.0% vs. 16.7%; *p* = 0.003) [41]. Altogether, 203 patients with coronary artery disease were analyzed and the results remained statistically significant after adjustment for clinical characteristics. A study by Schuurman et al. included 275 patients with 4 years follow-up and showed that each increase in LCBI by 100 units significantly increases the rate of MACE (HR = 1.19; 95% CI: 1.07–1.32, *p* = 0.001 for multivariate analysis) [42]. Howbeit, both of those studies were single center studies with small populations and short imagined segments were analyzed. Those obstacles were overcome by an LRP study, which enrolled 1563 patients, out of whom 1271 were allocated to 2 years follow-up [43]. In this study, non-stented segments were imagined with the pullback greater than 50 mm. The study showed that, in both patient- and plaque-level analysis, maxLCBI_4mm_ ≥ 400 was a strong predictor of NC MACE. NIRS imaging was also used in drug trials. In a YELLOW trial, 86 patients with obstructive CAD were recruited [44]. Patients were divided into two groups depending on the dose of the statin therapy. Although no changes in coronary plaque volume were noted after 7 weeks follow-up in those receiving a higher dose of statin, NIRS showed a significant reduction in LCBI_4mm_ (reduction—32.2% vs. −0.6%; *p* = 0.02).

Notably, ACS may also be caused by plaques with little or no amount of lipid content [9]. This phenomenon cannot be by any means predicted by NIRS alone; thus, the idea of using both NIRS and IVUS in one probe emerged. A recently published PROSPECT II study used such a combined modality [45]. Moreover, combining both NIRS and IVUS may allow for the accurate identification of PR, PE and CN—diagnosis so far reserved for OCT [46]. Those two studies are beyond the scope of this chapter and are described in more detail later on.

It should be remembered that LRP identification may not only predict future cardiac events but is also helpful during PCI procedure in order to avoid stent edge dissection or for assessment of periprocedural risk. Stone et al. showed that NIRS, by recognition of LRP, can identify lesions with increased periprocedural likelihood of periprocedural MI after stent implantation [47].

### 2.4. Optical Coherence Tomography (OCT)

Optical Coherence tomography utilized near-infrared light (Nir) to present the coronary vessel wall and coronary plaques. Since it utilizes light, as opposed to ultrasound for IVUS, it provides high-resolution images (10 µm–20 µm), which reflect the vessel structures in vivo with unpreceded precision [48,49]. Therefore, OCT seems to be the gold standard to present the traits of vulnerable plaque in vivo [50]. The imaging is performed in a similar way to IVUS using the disposables imaging catheters and a workstation to review images, guide imaging and analyze its results.

**Figure 4:**
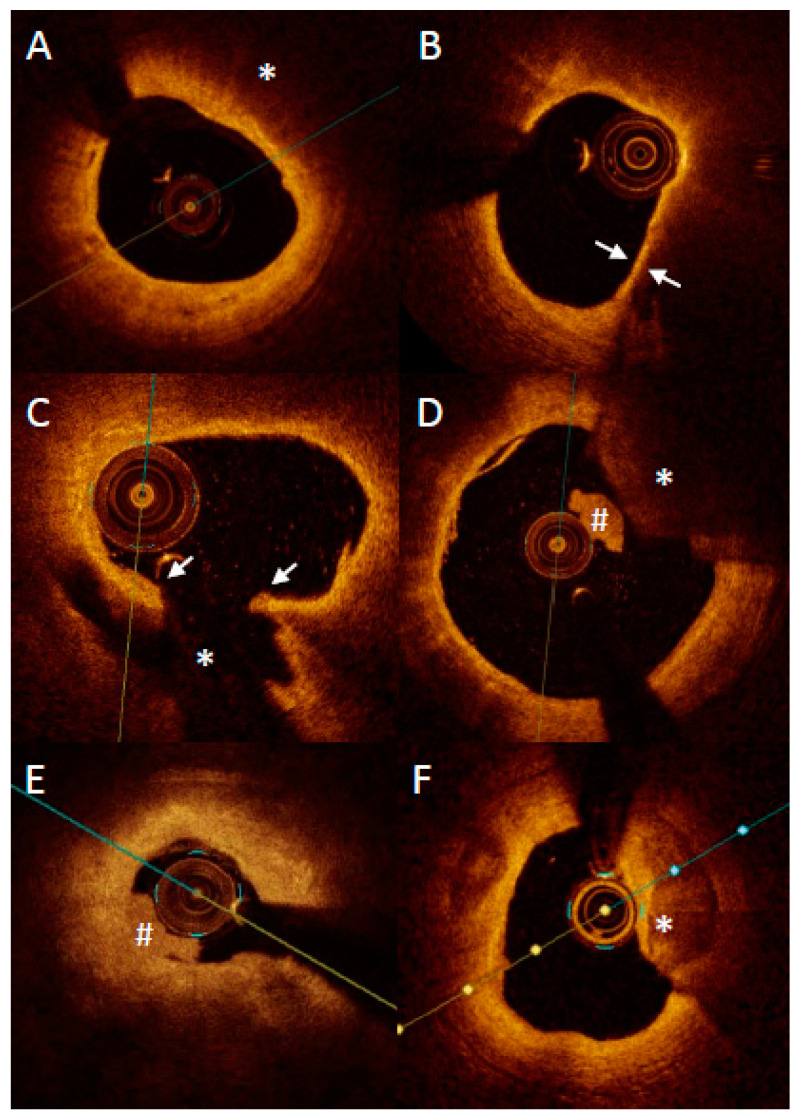
Representative images of optical coherence tomography findings in patients with acute myocardial infarction. Lipid plaque is characterized as signa poor regions (asterisk) with overlying signal-rich bands (**A**). Thin-cap fibroatheroma is defined as a lipid plaque occupying more than >90° in circumference and with fibrous cap thickness (arrows) less than a set threshold (usually 65 μm or 80 μm) (**B**). Plaque rupture is defined as disruption of fibrous cap (arrows) with visible cavity within the plaque ((**C**) asterisk). Red thrombus is described as highly backscattering structure with high attenuation ((**D**) asterisk), whereas white thrombus is less backscattering and has lower attenuation ((**D**,**E**) #). Erosion is described as presence of attached thrombus (usually white; #) overlying an intact and visualized plaque (**E**). Calcification protruding to the lumen is described as calcific nodule ((**F**) asterisk).

OCT with high accuracy describes the plaque composition, distinguishing between lipid-rich, fibrotic and calcified tissues (Figure 4). Lipid-rich plaques characterize a high signal attenuation in the vessel wall without clearly identified borders. A calcified lesion characterizes a high signal drop with clear borders. Fibrotic lesion characterizes a moderate signal attenuation with visible media behind the plaque [51].

Furthermore, it enables the measurement of the thickness of the fibrous cap covering the lipid pool and thus detects thin fibrous cap atheroma (TCFA). The globally accepted definition of TCFA is the lipid-rich lesion that extends to more than 90 degrees in the vessel circumference, covered with a fibrous cap less than 65 µm thick [38]. The autopsy study presented that lipid-rich lesions with fibrous cap less than 54 µm were mostly responsible for plaque rupture and sudden cardiac deaths events [5]. For OCT, the TCFA thickness threshold is 65 µm, concerning OCT resolution, which is around 10 µm. The downside of the OCT analysis is that it relies on the observers’ experience, and sometimes TCFA might be mistaken with massive calcification within the vessel, and with artifacts, such as signal drop [52]. OCT also enables the detection of other vulnerable plaque traits such as macrophages infiltration as bright spots scattered within the lipid-rich pools, as well as neovascularization [53,54].

In addition to the TCFA identification, the OCT is enabled to identify plaque erosion, which is a cause of the myocardial infarction in about 25–40% of all cases. Plaque erosion is defined as endothelial denudation [55]. Since the endothelial thickness (5 µm) is below the OCT resolution, Nir does not visualize it directly. However, a visible thrombotic mass attached to the vessel wall, in absence of a visible fibrotic cap rupture, is suggestive of plaque erosion. Another high-risk risk trait of atheroma detectable by OCT is calcification nodule. It is a calcification spot that sticks sharply to the lumen and exposes the vessel wall for intravessel thrombosis [55]. Further, OCT with its high resolution allows for the categorization of calcification in even more detail in patients with ACS: superficial calcific sheet, eruptive calcified nodules and calcified protrusion, with the first one being most frequent and being associated with the greatest postintervention myocardial damage [10].

OCT high-resolution images reflect the history of plaque formation. OCT easily enables the identification of plaque rupture, which is presented as a disrupted cap covering the lipid pool. It also detects the silent consequence of the plaque rupture, which is the healed plaque (honey-like structure with the signal shadowing within the plaque) [56]. Importantly, healed plaques are a quite frequent finding and may be associated with panvascular vulnerability. Russo et al., in a study comprising 163 patients with stable angina, found that healed culprit plaques in coronary arteries were present in more than half of the patients (53.4%) [4]. What is more, patients with healed culprit plaques showed more multivessel disease and had more features of plaque vulnerability. Similar results were found in ACS patients where over one quarter of them had healed plaques in culprit lesions [57]. Finally, Usui et al. showed that the presence of untreated healed plaques was positively correlated with non-culprit lesion related MACE [58].

Interestingly, OCT not only visualizes the images of the high-risk plaques but also provides information about the vessel healing after the stent identification. Since the new formation of intima (neointima) within the implanted stent may be impaired, the OCT detects it in vivo. The main high-risk neointima feature is the identification of noeatherosclerosis. OCT detects the newly formed lipid pool inside of the stent. Furthermore, just like in native lesions, OCT detects the in-stent lipid pool covered with a thin fibrous cap (<65 µm), which resembles the TCFA [59]. Such lesions may also rupture and be responsible for late-stent thrombosis.

OCT imaging lets us follow the healing pattern after the stent implantation. It distinguishes the homogenous and heterogenous neointima. Although the homogenous one is the result of the appropriate healing, the heterogenous may appear as the layered one, and the honey-like one and is responsible for future adverse events within the implanted stent [60].

The limitations of the OCT vulnerable plaque imaging are related to the features of the Nir. The application of Nir for imaging results in two main obstacles of OCT. Firstly, the high-resolution images are at the cost of signal penetration into the vessel wall. Therefore, the OCT is unable to provide the plaque burden, the key parameter for vulnerable plaques for IVUS. Secondly, the blood must be removed at the time of vessel imaging, because the Nir does not penetrate through the hemoglobin. It is achieved by the application of the contrast to the vessel during the OCT imaging. If the contrast cannot be applied appropriately the image quality is very poor and hampers the atheroma assessment. Therefore, ostial lesions and very tight lesions are extremely hard to visualize by the OCT, because the appropriate contrast application and thus complete blood removal at the time of imaging is very difficult [49].

#### The Impact of OCT Finding on Patients’ Risk

**Figure 5:**
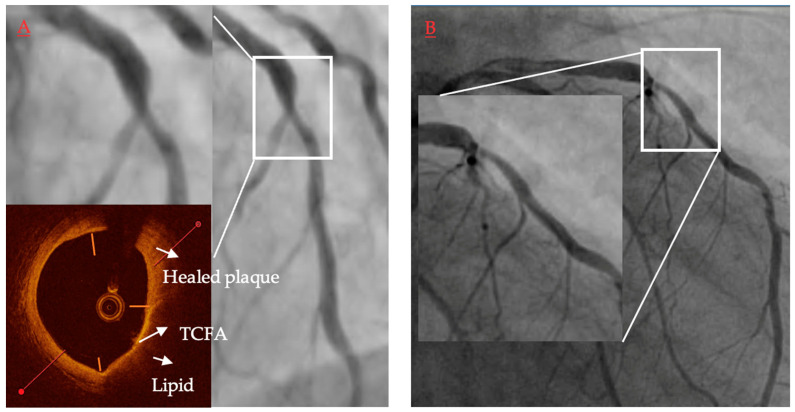
Representative case of vulnerable plaque progression. (**A**) Thin-cap fibroatheroma (TCFA) located in a short lesion in left anterior descending, diameter stenosis 62%, FFR baseline 0.86. Presence of a TCFA 3–6 o’clock adjacent to a healed plaque 12–3 o’clock. (**B**) Same lesion 13.5 months later when patient presented with non-ST-elevation myocardial infarction and underwent revascularization.

The initial studies presented the identification of long lipid and severely vessel narrowing lesions on OCT-identified patients at increased risk of MACE [61]. Recently, a published study, a COMBINE OCT-FFR trial, presented that the identification of TCFA in diabetic patients increased almost five times the risk of MACE at 18-months follow-up despite the absence of ischemia (Figure 5) [38]. This was the first trial shifting the concept of patients’ risk stratification from ischemia to plaque morphology. Importantly, a new subanalysis shows that not any lipidic plaque but only TCFA is related to future MACE, while ThCFA has very benign outcomes comparable to non-lipidic plaques. Comparable results were presented by Kubo et al. in a prospective study in which only lipid plaques were the cause of ACS during 6 years follow-up [62]. What is more important, ACS arose more frequently from lipid plaques with TCFA as compared to those with ThCFA (19% vs. 2%; HR 10.41 (95% CI: 6.48–16.73). Moreover, lipid-rich plaques (defined as lipid arc > 180 degrees) were also independent predictors of ACS. When combing both lipid-rich plaque and TCFA, those two features of plaque vulnerability were present in one third of all plaques, which caused ACS during the follow-up period. Intrudingly, macrophages were not predictors of future ACS events. On the contrary, in a CLIMA study, not only lipid-rich plaque and TCFA were predictors of major coronary events but also macrophages were associated with increased cardiac death or target vessel myocardial infarction. Discrepancy in those studies in regard to macrophages may be explained by subjectivity in the detection of macrophages and different criteria for cardiac endpoints. Regardless, it should be emphasized that macrophages are correlated with inflammation, which was found to be strongly associated with plaque vulnerability both in pathological and in vivo studies.

The question remains what to do with the identified TCFA in those patients. There are two options on the horizon: stenting it or applying more aggressive lipid-lowering therapy. According to current clinical practice, patients with non-ischemic LRP-TCFA lesions do not undergo coronary revascularization. Howbeit, as shown in numerous studies, contemporary medical therapies actually fail to prevent future adverse events in a considerable number of these patients [23,37]. While in current medical practice aggressive systemic medical treatment with novel, more potent cholesterol-lowering drugs is the most appropriate approach in these patients, yet, as of now, large-scale OCT studies about the effectivity of these drugs in stabilizing plaques and improving lesion composition are scarce [63,64].

Theoretically, considering the low MACE rate of OCT-guided focal stenting as compared to the high MACE rate under medical treatment, future clinical trials might assess even the usefulness of plaque sealing by focal percutaneous coronary treatment. Such a strategy was already studied in the PROSPECT-ABSORB trial, using guidance by intracoronary imaging with IVUS-NIRS [65]. In theory, OCT can be used to identify patients who might benefit from this novel strategy, and the use of stringent OCT-based criteria to guide potential treatment could reduce the number needed to treat by identifying and excluding patients in whom treatment benefit is unlikely. While sealing non-ischemic LRP-TCFA lesions with current generation drug-eluting stents is not recommended in current international guidelines, novel stents, scaffolds or other therapeutic options may emerge for this indication. Then, the percutaneous OCT-guided approach for treating non-ischemic LRP-TCFA lesions may represent an appealing novel strategy for assessing the safety and clinical efficacy of such therapeutic options in the clinical setting. On the contrary, as shown in recent studies, positive predictive value for MACE coming from vulnerable plaques is very low—in a study by Xing et al., 145 lesions would have to be treated to prevent two cases of MI [61,66].

A currently ongoing COMBINE-INTERVENE trial should further explore this hypothesis and help to answer the question of whether stenting non-ischemic lesions with features of plaque vulnerability may reduce MACE. In this trial, patients with multivessel disease will be randomized to: (1) FFR guided or (2) FFR and OCT guided arms. In FFR, guided arm revascularization will be conducted according to current clinical guidelines (with the 0.80 cut-off value for FFR). However, in the FFR and OCT, arm lesions with FFR equal or smaller than 0.75 as well as lesions with FFR greater than 0.75 but with features of plaque vulnerability will be stented. Hence, the COMBINE-INTERVENE trial should reduce MACE in several ways: (a) treating only severe ischemic lesions as guided by FFR; (b) treating the vulnerable plaque; (c) using OCT both before and after the stenting procedure for PCI optimalization.

Not only TCFA but also plaque erosion was studied. The erosion study presented that if OCT identifies the plaque erosion as the cause of myocardial infarction the anti-thrombotic therapy without stenting may be enough, and thus stenting for MI may not be required in this group of patients [67,68]. However, it should be kept in mind that plaque erosion identification mostly relies on observers’ experience and only trained analysts may accurately detect plaque erosion.

While TCFA remains the cornerstone feature of vulnerable plaque studies, other plaque morphological characteristics, such as healed plaque, have recently also evolved as being at risk for future adverse events [61,69]. Recent insights from the COMBINE trial show that TCFA lesions that progressed to MACE were frequently located adjacent to healed plaque within the same lesion. Treatment modalities for this type of plaque remain to date unknown.

Last but not least, it should be remembered that intravascular modalities are not only used for assessment of plaque vulnerability but also one of their primary goals was to improve the results of percutaneous coronary interventions. The use of both OCT and IVUS was shown to decrease the incidence of adverse events after coronary artery stenting and is widely used in different clinical settings (Table 2) [70].

In summary, NIRS allowed for the detection of lipid plaque, which was associated with greater incidence of feature MACE in numerous studies. However, the main limitation of NIRS is that it does not show the image of the plaque itself. Thus, the idea of merging NIRS and IVUS probes together emerged. The very high resolution of OCT allowed for detailed in vivo plaque analysis including the prevalence of macrophages, TCFA or calcification. Studies with the use of OCT allowed the distinguishment of plaques with the greater probability of progression, which then may lead to ACS. Currently ongoing OCT trials should answer the question of whether stenting not-significant coronary plaques, which have features of plaque vulnerability, may decrease the event of feature MACE.

### 2.5. OCT vs. VH-IVUS and NIRS

The first modality to detect TCFA in vivo was the HV-IVUS. The comparison between the two showed a lot of high false-positive values for VH-IVUS to detect OCT-identified TCFA [71]. However, the comparison of the two markedly increased the identification of TCFA itself [22]. The comparison between OCT and combined NIRS-IVUS images showed that OCT-derived TCFA characterized greater plaque burden and positive vessel remodeling [67]. The comparison between OCT and NIRS in the detection of lipid plaques showed a 20% difference in lipids detection in favor of NIRS [72]. However, we should keep in mind that only OCT enables the detection of TCFA in vivo, as opposed to IVUS and NIRS.

### 2.6. Fused Imaging

#### 2.6.1. Concept of Vulnerable Plaque in Fusion Imaging

Over the past few decades, many attempts have been made to define and predict the direction of vulnerable plaques and to understand the relationship between vulnerable plaques and vulnerable patients. Unfortunately, none of the stand-alone imaging tools used in daily clinical practice can predict with high accuracy the moment of plaque rupture [23,25,35,73,74]. These results have forced clinicians to redefine vulnerable plaque and the mechanisms of plaque rupture. It has been established that plaque instability arises from a complex interaction of anatomical and hemodynamic factors, such as microcalcifications, cholesterol crystals, macrophage apoptosis and endothelial shear stress (ESS). As the concept of atherosclerotic plaque has evolved, more metrics to determine plaque stability have been defined, leading to the development of new imaging techniques, methodologies and image processing algorithms to extract risk features. The diverse nature of coronary disease progression has pointed the way to the development of multimodal intravascular imaging techniques that combine two or more complementary modalities in a single catheter. This chapter presents the current state of research on intravascular image fusion in the detection of vulnerable atherosclerotic plaques and a summary of the studies is provided in Table 3.

#### 2.6.2. NIRS-IVUS Imaging

The first combined technique that had the potential to detect ruptured plaques was NIRS-IVUS. It was based on the concept that the destabilization and rupture of an atherosclerotic plaque is due to structural causes, i.e., a large, centrally located lipid core within the plaque.

Despite the important information about the presence of the lipid pool, the NIRS chemogram does not visualize the vessel structure and the location of the necrotic core within the plaque, which is a key determinant of plaque vulnerability. These limitations have been overcome by the addition of IVUS, which provides the ability to measure plaque structure. NIRS-IVUS fusion allows simultaneous visualization of the plaque structure and quantification of the presence of the lipid pool in the region of interest (ROI). The accuracy and sensitivity of NIRS-IVUS imaging has been confirmed by histopathological studies [75,76,80,81].

The COLOR registry demonstrated the association of LCBI with vulnerable atherosclerotic plaque [75]. The lipid-rich plaque study identified patients and coronary segments at risk of future major adverse coronary events [43,82]. A higher MACE risk was similarly associated with higher maxLCBI_4mm_, as in the COLOR study. Another prospective clinical trial currently underway investigating the potential of IVUS-NIRS in identifying MACE-prone atherosclerotic plaques is PROSPECT II. Several other studies using NIRS-IVUS have also confirmed the clinical application of this tool in identifying high-risk plaques in a non-culprit vessel segment [26,41,43,83].

NIRS-IVUS systems have been improved over the past few years and now exist in the form of a dual-frequency, dual-modal system (TVC Imaging System™ and Makoto Intravascular Imaging System™, Infraredx Inc., Bedford, MA, USA). The 3.2F rapid exchange catheter has a 2.4F entry profile and a 3.6F stem profile and is compatible with 6F guide catheters. This catheter pulls back at speeds of 0.5, 1.0 or 2.0 mm/s and rotates at 1800 rpm with a maximum imaging length of 15 cm, acquiring up to ~130,000 NIRS per 100 mm [84]. This is the first combined tool to be approved by the US Food and Drug Administration (FDA) to aid in the detection of high-risk atherosclerotic plaques.

However, despite all these successes, the main limitations of the NIRS-IVUS tool are the loss of signal behind calcified tissue and the low resolution, which is not sufficient to assess cap thickness and lumen boundary definition in the presence of thrombus.

#### 2.6.3. IVUS-OCT Imaging

Co-registration of IVUS and OCT is another promising multimodality imaging technique that has been developed to identify vulnerable plaques based on the concept that fibrous cap thickness, microcalcifications, cholesterol and macrophages are hallmarks of high-risk plaque [56,85,86,87,88,89,90,91].

In addition, several previous studies have reported and validated the usefulness of the combined use of IVUS and OCT in detecting vulnerable atherosclerotic plaques [92], comparing the results obtained with the single use of IVUS and OCT probes [17,77,93,94]. The first postmortem validation study showed that the positive predictive values for TCFA from stand-alone IVUS and OCT were 41% and 19% respectively, increasing to 69% when both modalities were used in combination [17]. The IVUS-OCT catheter allows simultaneous assessment of lipid-rich atherosclerotic plaques, bifurcations and deeply embedded tissue identified by IVUS images, whereas calcifications, stent struts and small dissections are more clearly identified by OCT imaging [95]. Currently, two companies, CONAVI (The Novasight HybridTM System Conavi Medical Inc., Toronto, ON, Canada) and TERUMO (The Dual Sensor hybrid IVUS-OCT, Tokyo, Japan), have integrated IVUS and OCT into a single catheter system, as very well described in [96], with the Novasight System now FDA-510(k)-approved and Health-Canada-approved. Future studies are expected to confirm the effectiveness of combined IVUS-OCT imaging catheters in detecting vulnerable atherosclerotic plaques.

#### 2.6.4. NIRS-OCT IMAGING/NIR(A)F-OCT Imaging

Hybrid NIRS and OCT/NIR(A)F and OCT have been proposed to improve outcomes after coronary stenting as a response to the PROSPECT study, which showed that the MACCE rate at 3.5 years was 12.9% after stenting caused by stented site and 11.6% due to non-stenotic vulnerable plaque. In addition, two separate reports identified thin caps over neovascularization arising inside stents using both OCT and INIRS imaging [59,97]. These results suggest that combined OCT-NIRS imaging in pre-existing stents may overcome the limitations of NIRS-IVUS imaging in identifying neoatherosclerosis [80].

NIR(A)F uses tissue fluorescence/autofluorescence to identify the molecular content of plaque composition [98]. It has been suggested that the complementarity of these methods allows lipid plaque localization and assessment as chemical/molecular information by NIRS/NIR(A)F and plaque thickness measurement, detection of macrophage deposition, by OCT [99,100]. NIR(A)F-OCT catheters are like a single OCT catheter [100].

The first human study of combined NIRAF-OCT was reported by [78] and showed that high NIRAF signal correlated with OCT-defined high-risk morphological features, such as TCFA, cap disruption and macrophage accumulation. In contrast, NIRAF signal was negative or low in plaques with a low-risk microstructural phenotype [101].

Moreover, ex vivo NIRS-OCT studies of human coronary arteries showed a relationship between the shape of the absorption spectrum and lipid-rich plaques using a prototype OCT-NIRS catheter developed at the Tearney Lab at Massachusetts General Hospital, Boston MA [102].

Further research is needed to confirm the utility of these techniques, and to develop advanced algorithms to overcome the limitations of NIR(A)F -OCT by compensating for fluorescence signal attenuation, optical scattering and tissue absorption.

#### 2.6.5. NIRF-OCT-IVUS Imaging

In a tri-modal IVUS/OCT/NIRF imaging system, NIRF imaging is used to detect the inflammatory response, IVUS to provide structural information about the full thickness of atherosclerotic plaques and OCT to extract depth information for NIRF, cap thickness, macrophage deposition and microscopic calcification.

Two ex-vivo studies have been conducted to validate the tri-modality system. The first experiment showed representative tri-modality images using Cy 5.5 [79]. This study confirmed the above assumptions that IVUS images show the entire structure of the vessel wall, OCT images show a clearer layered structure of the vessel wall and the NIRF signal indicates an inflammatory area with Cy5.5 coupled to annexin V. The second experiment demonstrated representative tri-modality images from ICG obtained by tri-modal catheter diameter 3.9 Fr [103]. As before, IVUS and OCT provide structural and microstructural information, and the NIRF signal indicates the area with injected ICG.

The tri-modality imaging system with integrated imaging probe is the tool with the most potential for clinical application to detect vulnerable atherosclerotic plaque. Real-time co-registration of three separate images is crucial for clinical applications.

In summary, the novel concept of fused imaging was proposed to overcome the disadvantages of individual imaging modalities. Several catheters are used, which combine NIRS-IVUS, IVUS-OCT, NIRS-OCT, NIRAF-OCT and NIRF-OCT-IVUS. Currently, ongoing clinical studies should answer the question of whether those combined modalities may allow for better detection of plaque vulnerability as well as better outcomes after percutaneous coronary interventions.

### 2.7. Fusion of Coronary Angiography and IVUS/OCT in 3D Reconstructions

The fusion of intravascular imaging (IVUS and OCT) and X-ray angiography data enables the reconstruction of coronary artery geometry and the generation of 3D models that combine with CFD techniques to assess flow in the vessel and to study the influence of hemodynamic forces on plaque evolution [104]. Since Stone in his studies suggested that ESSs play a role in plaque vulnerability, several studies have developed this idea [105,106]. The PREDICTION study was the first prospective clinical trial to use a fusion of intravascular imaging and X-ray angiography to assess the distribution of ESSs and determine the prognostic value of ESSs in predicting plaque progression [35]. Another PROSPECT study showed that lesions with high-risk morphology that were exposed to low ESS were likely to progress and cause cardiovascular events. A retrospective fusion of IVUS, OCT and coronary angiography from the IBIS 4 study showed that low ESS and plaque features derived from VH-IVUS were predictors of disease progression and destabilization in native vessels [107]. However, the exact mechanisms by which ESS acts to trigger plaque rupture requires further investigation.

### 2.8. High-Frequency and Dual-Frequency IVUS

Recently, several engineering groups have developed a dual-frequency IVUS imaging system to integrate a conventional IVUS transducer (35 MHz) with an ultra-high-frequency IVUS transducer (90–150 MHz) in a single catheter as another potential tool to identify vulnerable plaque. Several studies have successfully achieved adequate resolution to assess thin caps, but in vivo studies need to be performed in the future to understand the real improvement in detection high risk plaque and well understand the potential suppression effects [108,109,110].

## 3. Summary and Future Perspectives

**Figure 6:**
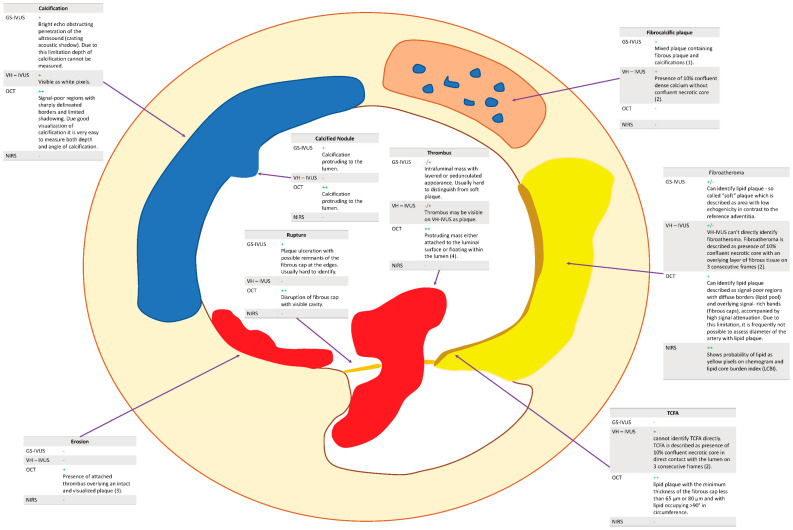
Advantages and disadvantages of respective intravascular modalities. GS-IVUS, gray-scale intravascular ultrasound; NIRS, near-infrared spectroscopy; OCT, optical coherence tomography; TCFA, thin-cap fibroatheroma; VH-IVUS, virtual histology intravascular ultrasound. For (1), (2) and (3), see the description of Table 4.

Different methods of intravascular imaging allow for the detailed in vivo assessment of coronary plaques (Table 2, Figure 6). Currently, those modalities are used in daily clinical practice in order to optimize PCI procedures as well as assess coronary arteries in patients presenting with MINOCA. Moreover, we know from numerous studies that even non-significant lesions with vulnerable plaque features such as lipid-rich plaque and TCFA are well-established predictors of future cardiac events. However, those studies did not address the question of what action should be taken for such lesions and thus there are no specific guidelines regarding the treatment of non-significant plaques with markers of plaque vulnerability. Ongoing and future studies will hopefully answer questions regarding: (1) the mechanical stabilization of non-significant coronary plaques showing features of plaque vulnerability; and (2) if patient-tailored therapy including novel drugs will decrease MACE in patients with coronary artery disease.

Not without significance is the fact that none of the currently used modalities can assess all known characteristics of plaque vulnerability; hence, the idea of combining different intravascular imaging methods has emerged. In current clinical practice, only the combination of NIRS and IVUS is commonly used; however, other combinations of intravascular modalities may become crucial to better characterization of vulnerable plaque and even more precise optimalization of coronary interventions.

Although not part of this review, recently non-invasive coronary imaging has been proposed as a modality to assess plaque vulnerability and in the near feature it may pose as a compelling alternative to invasive imaging [111].

## Figures and Tables

**Table 1:** Summary of studies assessing components of vulnerable plaque.

Authors/Study/Publication Year	Modalities	Study Size	Study Objective	Main Results	Main Limitations
Rodriguez-Granillo, 2005 [36]	IVUS-VH	55 patients	To assess the prevalence of intravascular ultrasound (IVUS)-derived thin-cap fibroatheroma (IDTCFA) and its relationship with the clinical presentation using spectral analysis of IVUS radiofrequency data. Definition of IDTCFA lesions—focal, necrotic-core-rich (≥10% of the cross-sectional area) plaques being in contact with the lumen, percent atheroma volume (PAV) ≥40%.	IVUS-VH identified IDTCFA as a more prevalent finding in ACS than in stable angina patients. ACS patients had a significantly higher incidence of IDTCFA than stable patients (0.7 (IQR 0.0 to 1.3) IDTCFA/cm vs. 0.2 (IQR 0.0 to 0.7) IDTCFA/cm, *p* = 0.031).	The lack of a direct comparison between IVUS-VH and histopathology
Stone, 2011 [23] PROSPECT [NCT00180466]	IVUS-VH	697 patients (313 had TCFA)	To confirm that ACS arise from atheromas with certain histopathological characteristics, and that these characteristics are not necessarily dependent on the degree of angiographic stenosis at that site.	In multivariate analysis, the authors found that plaque burden ≥70%, TCFA and minimal lumen area ≤4.0 mm^2^ were independent predictors of non-culprit lesion related major adverse cardiac events in lesion-level analysis. Importantly, the rate of MACE increased from HR 3.90 (95% CI, 2.25–6.76) with TCFA alone to HR 11.05 (95% CI, 4.39–27.82) when combining all of the described plaque futures.	Only the proximal 6 to 8 cm of the coronary tree were examined. All 106 non-culprit lesions associated with recurrent events were evaluated with the use of baseline angiography, but only 55 of these lesions were seen on gray-scale ultrasonography and only 51 were seen on radiofrequency intravascular ultrasonography.
Calvert, 2011 [24] VIVA	IVUS-VH	170 patients	TCFA identified by VH-IVUS are associated with major adverse cardiac events (MACE) in individual-plaque or whole-patient analysis.	The study showed that VH-IVUS TCFA was associated with MACE. The non-culprit lesion factors associated with non-restenotic MACE included VHTCFA (hazard ratio (HR): 7.53, *p* = 0.038) and plaque burden >70% (HR: 8.13, *p* = 0.011). VHTCFA (HR: 8.16, *p* = 0.007), plaque burden >70% (HR: 7.48, *p* < 0.001) and minimum luminal area <4.0 mm^2^ (HR: 2.91, *p* = 0.036) were associated with total MACE.	The definitions of TCFAs in VH-IVUS did not exactly match the histopathological definitions. VH-IVUS tended to overestimate the number of TCFAs compared to histology, and some histological ThCFAs were classified as VHTCFAs.
Brown, 2015 [22]	IVUS-VH, OCT	258 ROI from 14 human hearts	The combination of VH-IVUS and OCT improves the identification of TCFA.	Combined VH-IVUS/OCT imaging markedly improved TCFA identification. The sensitivity, specificity and diagnostic accuracy for TCFA identification were 63.6%, 78.1% and 76.5% for VH-IVUS and 72.7%, 79.8% and 79.0% for OCT. Combining VH-defined fibroatheroma and fibrous cap thickness ≤85 μm over three continuous frames improved TCFA identification, with a diagnostic accuracy of 89.0%.	Small study size; small longitudinal mismatches between imaging modalities
Cheng, 2014 [25] ATHEROREMO	IVUS-GS, IVUS-VH	581 patients	To investigate the prognostic value of the in vivo detection of high-risk coronary plaques by intravascular ultrasound (IVUS) in patients undergoing coronary angiography.	The study showed that presence of TCFA in non-culprit coronary artery is associated with greater incidence of death and ACS at 1 year follow-up. The presence of TCFA lesions was significantly associated with the composite of death or ACS only (present 7.5% vs. absent 3.0%; adjusted HR: 2.51, 95% CI: 1.15–5.49; *p* = 0.021). TCFA with a plaque burden of at least 70% were associated with a higher MACE rate both in the first 6 months (*p* = 0.011) and after 6 months (*p* < 0.001) of follow-up, while smaller TCFA lesions were only associated with a higher MACE rate after 6 months (*p* = 0.033).	The relatively small number of endpoints did not allow for the evaluation of whether adding IVUS imaging to a prognostic model with conventional risk factors would result in improved risk prediction. Missing repeat intracoronary imaging with IVUS virtual histology.
Fuji, 2015 [17]	IVUS-GS, OCT	165 coronary arteries from 60 autopsy hearts	To assess the accuracy of optical coherence tomography (OCT), gray-scale intravascular ultrasound (IVUS), and their combination for detecting thin-cap fibroatheromas (TCFA). A total of 685 pairs of images of OCT and IVUS were compared with histology.	PPV increased from 41% to 69% after IVUS and OCT combination. The sensitivity, specificity, PPV, NPV and DA of the combined use of OCT and IVUS for characterizing TCFA using histology as a standard were 92%, 99%, 69%, 99% and 99%, respectively.	The low prevalence of TCFA in histology (2%) may affect the statistical power to assess the diagnostic accuracy of TCFA.
Prati, 2020 [37] CLIMA	IVOCT	1003	To explore the predictive value of multiple high-risk plaque features in the same coronary lesion (minimum lumen area (MLA), fibrous cap thickness (FCT), lipid arc circumferential extension and presence of optical coherence tomography (OCT)-defined macrophages).	At 1 year, the primary clinical endpoint was observed in 37 patients (3.7%). In a total of 1776 lipid plaques, presence of MLA < 3.5 mm^2^ (hazard ratio (HR) 2.1, 95% confidence interval (CI) 1.1–4.0), FCT < 75 µm (HR 4.7, 95% CI 2.4–9.0), lipid arc circumferential extension > 180° (HR 2.4, 95% CI 1.2–4.8) and OCT-defined macrophages (HR 2.7, 95% CI 1.2–6.1) were all associated with increased risk of the primary endpoint. The pre-specified combination of plaque features (simultaneous presence of the four OCT criteria in the same plaque) was observed in 18.9% of patients experiencing the primary endpoint and was an independent predictor of events (HR 7.54, 95% CI 3.1–18.6). OCT-based classification showed limited sensitivity (positive predictive value 19.4%), but high specificity (negative predictive value 96.9%) for the primary endpoint, and remained an independent predictor of 1 year events after correction for the other confounding variables.	The registry included patients with various clinical presentation and cardiovascular risk profiles uniquely pooled by the intraprocedural OCT assessment of proximal LAD. The combination of the four high-risk plaque features was uncommon.
Kedhi, 2021 [38] COMBINE	IVOCT	482	To study the impact of optical coherence tomography (OCT)-detected thin-cap fibroatheroma (TCFA) on the clinical outcomes of diabetes mellitus (DM) patients with fractional flow reserve (FFR)-negative lesions.	Among DM patients with ≥1 FFR-negative lesions, TCFA-positive patients represented 25% of this population and were associated with a five-fold higher rate of MACE despite the absence of ischaemia. The Cox regression multivariable analysis identified TCFA as the strongest predictor of major adverse clinical events (MACE) (hazard ratio 5.12; 95% confidence interval 2.12–12.34; *p* < 0.001).	

**Table 2:** Differences in IVUS and OCT in daily practice.

	IVUS vs. OCT	Comment
Assessment of non-calcified and non-LM coronary plaques before stent implantation	Equal	OCT may provide more information regarding plaque composition (for example lipid plaque and optimal stent edge placement).
Assessment of calcified and non-LM coronary plaques before stent implantation	OCT better	Calcification obstructs penetration of the ultrasound (casting acoustic shadow).
Assessment of LM coronary plaques before stent implantation	IVUS better	OCT may be used in non-ostial LM lesions provided proper blood removal.
Optimalization after stent implantation	OCT better	Images from OCT due to high resolution may be easier to interpret provided proper blood removal (not possible in LM ostial lesions).
Spontaneous coronary dissection	IVUS better or equal	OCT may provide easier interpretation of SCAD and is used in clinical practice; however, contrast flush may propagate SCAD.
Stent failure	OCT	Higher resolution and easier interpretation with OCT.
Neoatherosclerosis	OCT	Higher resolution and easier interpretation with OCT.
Imaging in setting of ACS	OCT	OCT may provide information regarding the mechanism of ACS including plaque rapture, erosion or calcified nodule.
CTO	IVUS	OCT requires contrast flush, which is not possible in CTO. Moreover, when using OCT, it is not possible to provide continuous visualization of one chosen coronary artery.
CKD stage 4	IVUS	OCT requires continuous contrast flush during pullback.

CKD, chronic kidney disease; CTO, chronic total occlusion; IVUS, intravascular ultrasound; LM, left main; OCT, optical coherence tomography; SCAD, spontaneous coronary artery dissection.

**Table 3:** Summary of studies with fused intravascular imaging.

Authors/Publication Year/Study	Fused Imaging Modalities	Study Size	Objectives	Main Results	Main Limitations
Goldstein [75], 2011 COLOR Registry [NCT00831116]	NIRS-IVUS	62	Prospective identification of LCP with catheter-based near-infrared spectroscopy (NIRS) may predict an increased risk of periprocedural MI and facilitate development of preventive measures.	The primary finding of the study is that in patients with coronary artery disease, PCI of lesions with a large lipid core (maxLCBI_4mm_ ≥ 500 by NIRS) is associated with a 50% risk of periprocedural MI (95% CI, 28–62), compared with only a 4.2% risk (95% CI, 0.8–11) for lesions without a large lipid core (maxLCBI_4mm_ < 500 by NIRS).	The number, type, timing and frequency of biomarker determination were not standardized. A small sample size.
Kini, 2013, [44] YELLOW [NCT01567826)]	NIRS-IVUS	86 patients	To determine the impact of short-term intensive statin therapy on intracoronary plaque lipid content.	The median reduction (95% confidence interval) in LCBI_4mm_ max was significantly greater in the intensive versus standard group (−149.1 [−210.9 to −42.9] vs. 2.4 [−36.1 to 44.7]; *p* = 0.01). Short-term intensive statin therapy may reduce lipid content in obstructive lesions.	A small sample size and short duration of follow-up. The baseline LCBI was significantly higher in patients randomly allocated to intensive versus standard therapy.
Puri [76] 2015	NIRS-IVUS	116 coronary arteries of 51 autopsied hearts	To assess the relationships between intravascular ultrasound (IVUS)-derived PB and arterial remodeling with near-infrared spectroscopy (NIRS)-derived lipid content in ex vivo and in vivo human coronary arteries.	Lesion-based analyses demonstrated the highest LCBI and remodeling index within coronary fibroatheroma (P trend < 0.001 and 0.02 versus all plaque groups, respectively). Prediction models demonstrated similar abilities of PB, LCBI and the remodeling index for discriminating fibroatheroma (c indices: 0.675, 0.712, and 0.672, respectively). A combined PB + LCBI analysis significantly improved fibroatheroma detection accuracy (c index 0.77, *p* = 0.028 versus PB; net-reclassification index 43%, *p* = 0.003).	Small study size and on autopsied heart
Waksman [43] 2019 LRP Study NCT02033694	NIRS-IVUS	1271 patients	To investigate the relationship between LRPs detected by NIRS-intravascular ultrasound imaging at unstented sites and subsequent coronary events from new culprit lesions.	The 2-year cumulative incidence of NC-MACE was 9% (*n* = 103). The unadjusted hazard ratio (HR) for NC-MACE was 1.21 (95% CI 1.09–1.35; *p* = 0.0004) for each 100-unit increase in maxLCBI_4mm_ and the adjusted HR was 1.18 (1.05–1.32; *p* = 0.0043). In patients with a maxLCBI_4mm_ over 400, the unadjusted HR for NC-MACE was 2.18 (1.48–3.22; *p* < 0.0001) and the adjusted HR was 1.89 (1.26–2.83; *p* = 0.0021).	
Terada [46], 2021 PROSPECT II	NIRS-IVUS, OCT	244 patients	To investigate the ability of combined near-infrared spectroscopy and intravascular ultrasound (NIRS-IVUS) to differentiate plaque rupture (PR), plaque erosion (PE) or calcified nodule (CN) in acute myocardial infarction (AMI).	NIRS-measured maxLCBI_4mm_ was significantly largest in OCT-PR (705 (interquartile range (IQR): 545 to 854)), followed by OCT-CN (355 (IQR: 303 to 478)) and OCT-PE (300 (IQR: 126 to 357)) (*p <* 0.001). The NIRS-IVUS classification algorithm using plaque cavity, convex calcium and max LCBI_4mm_ showed a sensitivity and specificity of 97% and 96% for identifying OCT-PR, 93% and 99% for OCT-PE, and 100% and 99% for OCT-CN, respectively.	Recognition of PR, PE and CN using OCT as a reference, without considering the intrinsic and insurmountable limitations of OCT technology. Aspiration thrombectomy and balloon angioplasty prior to imaging may have induced iatrogenic rupture of the fibrous cap and reduced the lipid composition of the PR.
Li [77] 2015	IVUS-OCT	50 human coronary arteries (in vitro)	To investigate the capability of recognition of vulnerable plaques using this IVUS-OCT technology.	Histology confirmed that TCFA and false TCFA can be differentiated using IVUS-OCT images. The full integration of the two complementary techniques of OCT and IVUS permits accurate evaluation of total plaque burden and plaque morphology by using an in vitro human cadaver study.	Limited study size and only on autopsied vessel
Ughi [78], 2016	OCT-NIRAF	12 patients	First clinical imaging of human coronary arteries in vivo using a multimodality OCT and near-infrared autofluorescence (NIRAF) intravascular imaging system and catheter.	High-quality intracoronary OCT and NIRAF image data (>50 mm pullback length) were successfully acquired without complication in all patients. In a substudy of 4 repeated pullbacks, NIRAF reproducibility was excellent with an average Pearson’s correlation coefficient of 0.925 ± 0.015.	Small study
Liang [79], 2014	NIRF-OCT-IVUS	-	The study presented a trimodality imaging system and an intravascular endoscopic probe for the detection of early-stage atherosclerotic plaques.	The first ex vivo imaging of a normal New Zealand white rabbit aorta in which two model plaques had been planted inside the blood vessel wall.	Large dimension of probe Long imaging time

**Table 4:** Advantages and disadvantages of respective intravascular modalities.

	GS-IVUS	VH-IVUS	NIRS	OCT
Fibroatheroma	Can identify lipid plaque—so-called “soft” plaque—which is described as the area with low echogenicity in contrast to the reference adventitia.	VH-IVUS cannot directly identify fibroatheroma. Fibroatheroma is described as the presence of 10% confluent necrotic core with an overlying layer of fibrous tissue on three consecutive frames (2).	Shows the probability of lipid as yellow pixels on the chemogram and lipid core burden index (LCBI).	Can identify lipid plaque described as signal-poor regions with diffuse borders (lipid pool) and overlying signal-rich bands (fibrous caps), accompanied by high signal attenuation. Due to this limitation, it is frequently not possible to assess the diameter of the artery with lipid plaque.
Calcification	Bright echo obstructing penetration of the ultrasound (casting acoustic shadow). Due to this limitation, the depth of calcification cannot be measured.	Visible as white pixels.	NA	Signal-poor regions with sharply delineated borders and limited shadowing. Due to good visualization of calcification, it is very easy to measure both the depth and angle of calcification.
Fibrocalcific plaque	Mixed plaque containing fibrous plaque and calcifications (1).	Presence of 10% confluent dense calcium without confluent necrotic core (2).	NA	NA
Calcific nodule	Calcification protruding to the lumen.	NA	NA	Calcification protruding to the lumen.
TCFA	GS-IVUS does not have a resolution high enough to visualize TCFA.	VH-IVUS cannot identify TCFA directly. TCFA is described as the presence of 10% confluent necrotic core in direct contact with the lumen on three consecutive frames (2).	NA	Lipid plaque with a minimum thickness of the fibrous cap of less than 65 μm or 80 μm and with lipid occupying >90° in circumference.
Erosion	NA	NA	NA	Presence of attached thrombus overlying an intact and visualized plaque (3).
Rupture	Plaque ulceration with possible remnants of the fibrous cap at the edges. Usually hard to identify.	NA	NA	Disruption of fibrous cap with visible cavity.
Thrombus	Intraluminal mass with layered or pedunculated appearance. Usually hard to distinguish from soft plaque.	Thrombus may be visible on VH-IVUS as plaque.	NA	Protruding mass either attached to the luminal surface or floating within the lumen (4).

GS-IVUS, gray-scale intravascular ultrasound; NIRS, near-infrared spectroscopy; OCT, optical coherence tomography; TCFA, thin-cap fibroatheroma; VH-IVUS, virtual histology intravascular ultrasound. (1) Intermediate echogenicity between soft (hypoechoic) plaque and highly echogenic calcified plaques. (2) Necrotic core on VH-IVUS is visible as red pixels, calcium is visible as white pixels. (3) OCT does not have resolution high enough to visualize erosion. (4) Red thrombus is erythrocyte-rich and is highly backscattering and has high attenuation whereas white thrombus is platelet-rich and is less backscattering and has lower attenuation.

## Data Availability

Not applicable.
